# Friction properties of a new silk fibroin scaffold for meniscal replacement

**DOI:** 10.1016/j.triboint.2017.01.038

**Published:** 2017-05

**Authors:** Daniela Warnecke, N.B. Schild, S. Klose, H. Joos, R.E. Brenner, O. Kessler, N. Skaer, R. Walker, M. Freutel, A. Ignatius, L. Dürselen

**Affiliations:** a Institute of Orthopaedic Research and Biomechanics, Centre for Trauma Research Ulm, Ulm University Medical Centre, Germany; b Division for Biochemistry of Joint and Connective Tissue Diseases, Department of Orthopaedics, Ulm University Medical Centre, Germany; c Centre of Orthopaedics and Sports, Zurich, Switzerland; d University Medical Centre, Clinic for Orthopaedic Surgery, Magdeburg, Germany; e Orthox Ltd. Abingdon, UK

**Keywords:** Friction, Silk, Meniscus replacement, Cartilage, Lubricin

## Abstract

The menisci protect the articular cartilage by reducing contact pressure in the knee. To restore their function after injury, a new silk fibroin replacement scaffold was developed. To elucidate its tribological properties, friction of the implant was tested against cartilage and glass, where the latter is typically used in tribological cartilage studies. The silk scaffold exhibited a friction coefficient against cartilage of 0.056, which is higher than meniscus against cartilage but in range of the requirements for meniscal replacements. Further, meniscus friction against glass was lower than cartilage against glass, which correlated with the surface lubricin content. Concluding, the tribological properties of the new material suggest a possible long-term chondroprotective function. In contrast, glass always produced high, non-physiological friction coefficients.

## Highlights

• A new silk fibroin scaffold provides low friction coefficient against articular cartilage.
• A high friction coefficient is observed when tested against glass due to the missing interstitial fluid load support.
• Friction studies of non-biological materials, e.g. for implants, should be carried out against articular cartilage.
• Meniscus has a higher lubricin content on its surface in comparison to articular cartilage.

## 1 Introduction

The semilunar menisci are located between the femoral condyles and the tibial plateau of the knee joint [1], [2], [3], [4]. They play a decisive role in load distribution by increasing the contact area between the incongruent femoral and tibial articular surfaces. Additionally, they are involved in secondary joint stabilisation, nutrient distribution and providing joint motion at low friction [3], [4], [5]. Loads of up to 3.5 times body weight are transferred through the knee joint during activities of daily life, whereby in general 45–70% of the total load is transmitted through the menisci [6], [7]. In experiencing such high mechanical stress, the menisci are prone to injuries requiring surgical intervention in approximately 85% of cases [8]. The most frequent surgical therapy for meniscus injuries is a partial meniscectomy. However, various studies have shown that a partial meniscectomy determines the onset of cartilage degeneration, leading to osteoarthritis (OA) in the long term [9], [10], [11].

Removing meniscal tissue leads to a reduced contact area associated not only with an increased contact pressure but also with greater friction [3], [4], [5], [10], [11], [12]. McCann et al. additionally identified fibrillation of the cartilage surfaces immediately after removal of meniscal tissue and wear of the articular cartilage [12]. Therefore, concepts for the restoration of meniscal function by implantable devices should comprise not only the ability to transmit loads but should also consider how to mimic the low friction provided by the native meniscus. Various biomaterials with different biomechanical properties have been developed to replace the injured meniscal tissue and restore its function [13], [14]. Two resorbable scaffolds (CMI® by Ivy Sports Medicine GmbH, Actifit® by Orteq Ltd.) are currently available and in clinical practice. However, Sandmann et al. showed their lack of biomechanical stability in comparison to the native meniscus within an *in vitro* study [15]. Another non-resorbable scaffold for partial meniscal replacement based on silk fibroin (FibroFix™ Meniscus, Orthox Ltd., Abingdon, UK) was recently investigated in a sheep model, showing promising results regarding biocompatibility and the prevention of OA after 6 months [16]. However, no results are currently available predicting its long-term chondroprotective function. To maintain the chondroprotective properties of a meniscal scaffold over an extended period of time, its frictional behaviour is of major importance as a high friction coefficient leads to wear, which is associated with cartilage fibrillation.

In general, friction is defined as the resistance of motion between two surfaces that are in contact. According to Coulomb, the friction force F_R_ is equal to the product of the friction coefficient *µ* and normal force F_N_. Therefore, the friction coefficient is a material property and could be calculated from the quotient of friction and normal force. However, within synovial joints, friction is much more complex. This is due to the biphasic viscoelastic nature of the opposing surfaces of the meniscus and articular cartilage lubricated by synovial fluid [17].

Various studies investigated the frictional behaviour of articular cartilage [18], [19], [20], [21], [22], [23], [24], [25], [26]. Regarding the three lubrication modes transferred from mechanical friction analysis (i.e., fluid, boundary and mixed lubrication), numerous tribology theories were postulated to describe the remarkable frictional behaviour of articular cartilage [17], [26], [27], [28], [29], [30], [31]. Within these studies, it has been shown that the friction in synovial joints is multifactorial and several parameters, including applied normal load and strain, sliding speed, time and lubricant, influence the friction mode [23], [24], [25], [26], [31]. Furthermore, the opposing surface used to test friction characteristics naturally has a major effect on the friction coefficient. Glass, which was typically used in most cartilage friction studies [19], [22], [25], [28], [32], provides a smooth counter surface, but its use appears at least debatable in terms of its lack of physiological properties. Testing articular cartilage against glass leads to an increase in friction over time until an equilibrium is reached due to the biphasic, viscoelastic nature of cartilage [33]. This phenomenon is most likely attributable to interstitial fluid pressurisation within the articular cartilage [19], [20], [22], [25], [34]. It has been shown that the applied load is initially supported by the fluid phase of the biphasic cartilage, resulting in a very low friction coefficient. Under a persisting load, the load support is continuously transferred to the solid matrix, resulting in an increasing friction coefficient [22], [34]. This phenomenon is typically observed in highly hydrated and biphasic tissue.

The above mentioned silk fibroin based scaffold for permanent meniscal replacement is processed into a porous matrix with a smooth surface [16]. Although its ultrastructure differs considerably from the native meniscus it showed promising results in a first *in vivo* trial in sheep [16]. Additionally, Parkes et al. demonstrated a cartilage-like friction response of silk protein hydrogels in articular cartilage repair [35]. Based on these findings, we hypothesised that the friction coefficient of the silk fibroin scaffold for meniscal replacement is comparable to physiologically articulating surfaces. We further hypothesised that glass as an opposing surface leads to higher friction coefficients not only for articular cartilage but also for meniscus and scaffold in comparison to those achieved when tested against cartilage.

## 2 Method

### 2.1 Study design

**Fig. 1:**
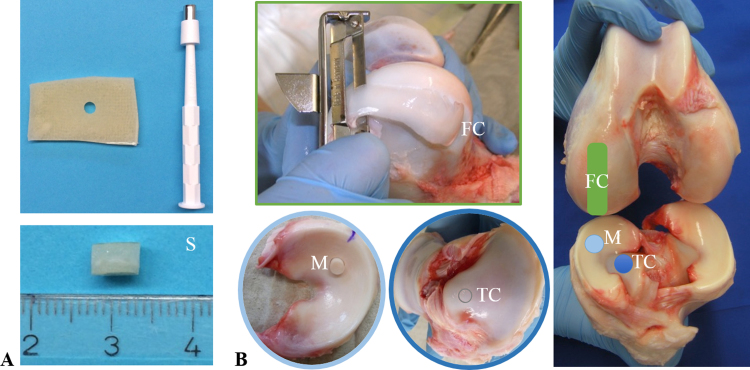
Cylindrical samples were prepared from flat sheets of the silk fibroin scaffold (S) (a), as well as being retrieved from the meniscus (M) and tibial cartilage (TC) of bovine knee joints (b). Flat cartilage samples were taken from the femoral condyle (FC) serving as the opposing surface during friction testing (b).

The frictional properties of the silk fibroin scaffold for meniscal replacement were determined in comparison to the physiologically articulating surfaces of meniscus and articular cartilage. Therefore, cylindrical samples were prepared from the silk fibroin scaffold as well as being retrieved from the meniscus and tibial cartilage of seven intact bovine knee joints (age: 3 months) (Fig. 1). Each cylindrical sample was first tested against a flat cartilage sample, which was also harvested from the bovine knee joints, using a *pin-on-plate* friction-testing device. During testing, the flat opposing surface slid cyclically against the cylindrical samples, while a constant normal load of 14.6 N was applied to them, resulting in a detectable friction force. All samples were stored overnight in phosphate-buffered saline (PBS) at 4 °C for recovery and tested against glass the next day to test the second hypothesis.

### 2.2 Detailed procedure

#### 2.2.1 Sample preparation

**Fig. 2:**
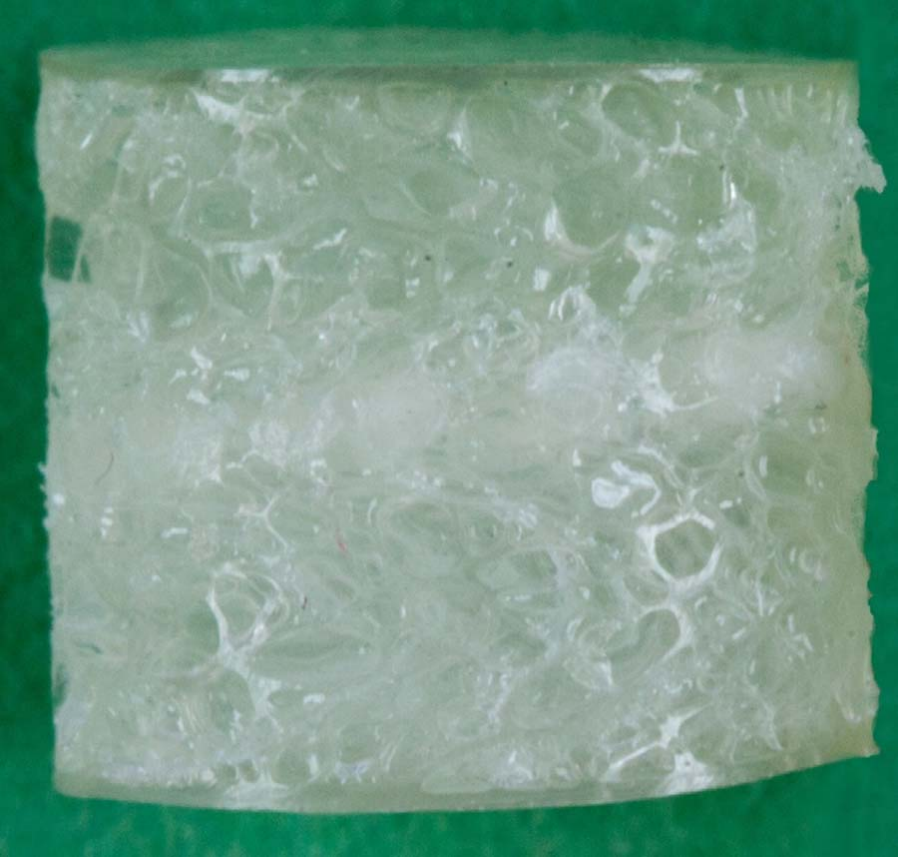
Macroscopic image of the meniscal silk fibroin scaffold (FibroFix™, Orthox Ltd., Abdindon, UK).

The scaffold samples used in this study were manufactured by Orthox Ltd. and consisted of a biomaterial based on the protein, fibroin, extracted from silk fibres of the mulberry silk moth *Bombyx mori*, which was subsequently processed into a porous matrix (Fig. 2). Seven scaffold samples were retrieved from 6 flat sheets (height h_0_=3.4 mm±0.6 mm) using a biopsy punch (ø=6 mm). Seven fresh intact bovine knee joints were ordered from a local butcher and stored at −20 °C. The day before testing, the joints were kept at 4 °C to thaw. Cylindrical meniscus samples (ø=6 mm) were punched out from the medial meniscus at the transition of the posterior horn and the *pars intermedia* perpendicular to the surface that was physiologically in contact with the femoral condyle. For later fixation in the friction testing apparatus, the cylindrical samples required parallel surfaces. Therefore, the distal region of the meniscus, which articulates with the tibial plateau surface, was removed using a microtome, resulting in specimens 2 mm in height. In addition, cylindrical osteochondral specimens were harvested out of the medial tibial condyle at the *eminentia intercondylaris*, perpendicular to the surface, using a trephine drill (ø=6 mm). The subchondral bone was removed and the cylindrical cartilage samples were cut, resulting in specimens approximately 2.5 mm in height. Flat cartilage samples were taken from each of the medial femoral condyle using a peeler (n=7). The flat cartilage samples served as an opposing surface during friction testing (approx. 40 mm length, 20 mm width and 2 mm height). To test the second hypothesis, an uncoated, smooth, glass microscope slide was used as the opposing surface (VWR* Plain Micro Slides, VWR International GmbH, Darmstadt, Germany). All specimens were maintained hydrated during preparation and were stored in PBS at 4 °C until testing.

#### 2.2.2 Friction-testing apparatus

**Fig. 3:**
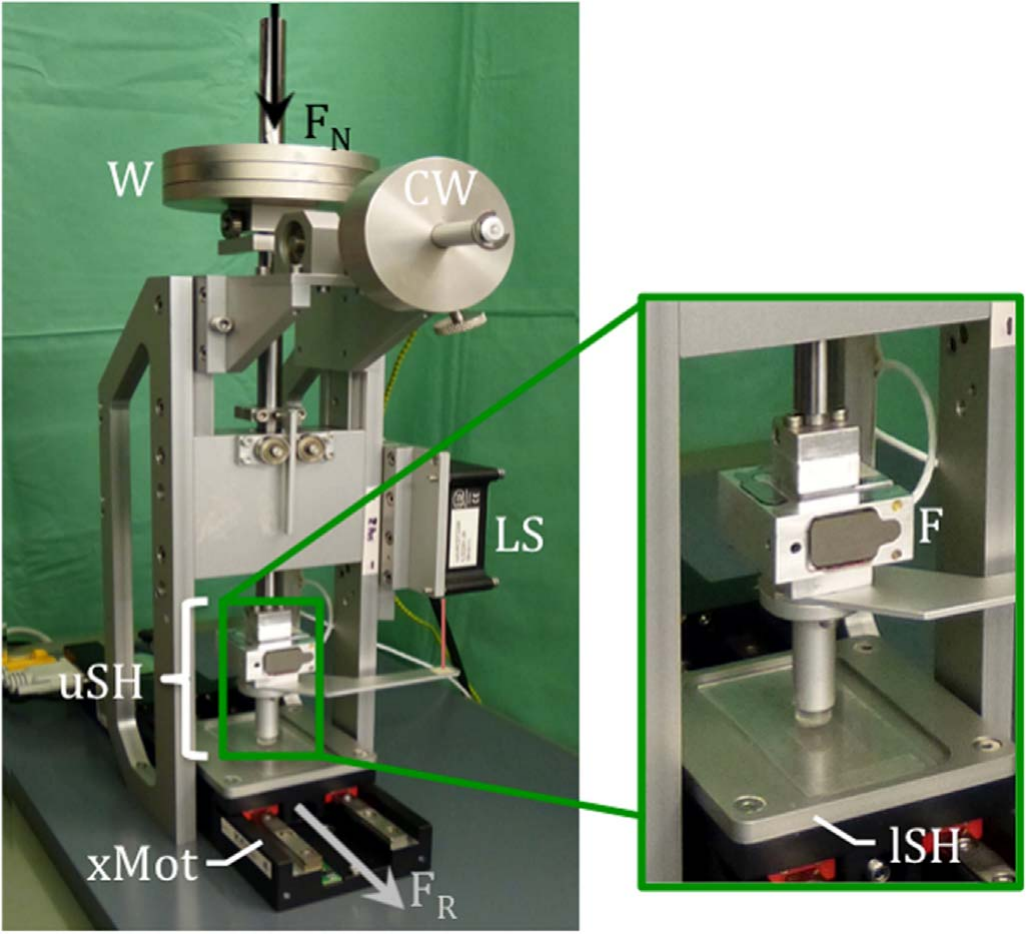
*Pin-on-plate* friction-testing device. A constant normal load (F_N_=14.6 N) was applied by placing weights (W) to the upper sample holder (uSH), on the end of which the cylindrical samples (meniscus M, silk fibroin scaffold S, tibial cartilage TC) were mounted. During testing, the lower sample holder (lSH) with the femoral cartilage (FC) or glass (G) sample was sliding against it, driven by a linear motor (xMot). A force sensor (F) recorded the resulting friction force F_R_, while a contactless laser distance sensor (LS) additionally measured the displacement signal of the cylindrical samples. Counter-weights (CW) served as prevention for unintentional load application due to the tare weight of the testing apparatus to the cylindrical samples.

To determine the friction coefficient of the meniscal scaffold in comparison to meniscus and articular cartilage, a *pin-on-plate* friction-testing apparatus was designed (Fig. 3). It consisted of an aluminium frame with an upper specimen holder (uSH), including a custom 3 DOF load cell (F) (F_x,y_=max 20 N, F_z_=max 50 N; accuracy class: 0.5%; ME-Meßsysteme GmbH, Henningsdorf, Germany) and an adjustable counter-weight (CW) to prevent an unintentional load application to the small sample mounted on the uSH due to the tare weight of the testing apparatus. At the upper end of the uSH, weights (W) were placed to apply a constant axial load F_N_. A computer-controlled linear motor (xMot) (M-404.4PD, Physik Instrumente PI GmbH & Co. KG, Karlsruhe, Germany) was mounted underneath the uSH carrying the sliding counter surface of femoral cartilage or glass, representing the lower sample holder (lSH).

Additionally, a contactless laser distance sensor (LS) (ILD 2220-20, MicroEpsilon, Ortenburg, Germany) was mounted on the aluminium frame, continuously recording the specimen deformation.

A custom-made LabVIEW program (LabVIEW, National Instruments, Austin, USA) was used to control the motor and for data acquisition.

#### 2.2.3 Testing protocol

Before each test, the cylindrical samples (FibroFix™ Meniscus scaffold: S, meniscus: M, tibial cartilage: TC) were fixed in the uSH of the friction-testing apparatus in randomised order. On the first experimental day, each sample was tested against the flat femoral cartilage sample as the opposing surface.

After starting the testing software, weights of approx.1.5 kg were placed on the uSH, inducing a constant load F_N_ of 14.6 N. This load corresponds to a contact pressure of 0.5 MPa, which is representative of light physiological loading conditions in quadrupeds during walking [36], [37]. The lower sample was slid against the uSH for 250 cycles at a velocity of 1 mm/s with a stroke length of±15 mm until the equilibrium was reached, resulting in a total testing time of 125 min. During testing, the friction force F_R_ and normal force F_N_ were continuously recorded from the 3 DOF load cell at a sample rate of 100 Hz.

On day two and after a recovery time of >12 h in PBS at 4 °C without any load application, the friction tests were repeated against glass, which was typically used as a counter surface in friction studies. The tests on both days were performed at a room temperature of approximately 20 °C and a humidity of approximately 34% using bovine synovial fluid as a lubricant. Here, special attention was payed that the samples were fully covered with lubricant throughout the entire testing period to prevent sample dehydration. Consequently, additional lubricant was provided when deemed necessary. All samples and biological materials were brought to room temperature before testing.

To evaluate the frictional behaviour, the friction coefficient at the beginning of the experiment (*µ*_*0*_) and after reaching the equilibrium (*µ*_*eq*_) as well as the strains (*ε*_*0*_ and *ε*_*eq*_) at the respective times was determined. The friction coefficients *µ*_*0*_ and *µ*_*eq*_ were calculated using the quotient of the friction force F_R_ and normal force F_N_ recorded during the first and the last three cycles of the experiment, respectively. The strains were determined by dividing the deformation recorded by the LS by the initial sample height h_0_.

### 2.3 Lubricin analysis

After reviewing the initial friction experiments, the results indicated that the friction coefficient at equilibrium was higher for articular cartilage than for meniscus when tested against glass. We speculated that a difference in the surface structure of the two tissues may be responsible for this. Because surface lubricin has been attributed an important role in low friction of joints [26], [38], [39], we decided to investigate the lubricin content on the surface of the meniscus and articular cartilage of additional bovine knee joints. Cylindrical meniscal samples (ø=6 mm) were taken from the anterior horn of the medial meniscus (n=10) while cylindrical tibial cartilage samples were obtained from both the *eminentia intercondylaris* (n=6) and the tibia plateau (n=6).

To detect the native form of bovine lubricin, 6.8 µm cryosections of the samples were cut and stained immunohistologically using a monoclonal mouse anti-bovine lubricin antibody (clone 3A4, MD Bioproducts, Zurich, Switzerland). As a secondary antibody, biotin-conjugated anti-mouse immunoglobulin was used as well as the detection kit LSAB-universal kit (K0690, DAKO, Hamburg, Germany) with horseradish peroxidise-conjugated streptavidin, as described by the manufacturer's protocol.

To quantify the amount of lubricin, the length of the positively stained tissue surface was determined using the image analysis software AxioVision 4.8.2 (Zeiss, Oberkochen, Germany) and related to the total length of the tissue in one histological slide for each specimen.

### 2.4 Statistics

All recorded and calculated friction data were classified into six groups (each n=7) depending on the material pairings: meniscal scaffold vs. femoral cartilage; meniscal scaffold vs. glass; native meniscus vs. femoral cartilage; native meniscus vs. glass; tibial cartilage vs. femoral cartilage; tibial cartilage vs. glass. As the friction coefficients for each group at the onset of the test (*µ*_*0*_) and after reaching the equilibrium (*µ*_*eq*_) as well as the strains at these two different time points (*ε*_*0*_ and *ε*_*eq*_, respectively) were normally distributed (normal probability plot, Shapiro-Wilk test [40]), the data were averaged. All further statistical analyses were performed using GraphPad Prism® software (GraphPad Software, Inc., La Jolla, USA).

To compare the frictional behaviour of all cylindrical samples depending on the opposing surface, one-factor analysis of variance (ANOVA) with uncorrected Post-Hoc Fisher's LSD tests with a single pooled variance were performed. To detect differences according to the opposing surfaces as well as to obtain information on the time-dependent changes in friction coefficients, two-factor ANOVA with repeated measures were conducted. Due to the testing procedure, there were two strain values per cylindrical sample, one measured when tested against femoral cartilage and one when tested against glass. To exclude any influence of the opposing surface on the strain, the difference between each cylindrical sample according to the opposing surface was determined by performing paired *t*-tests.

The amounts of lubricin determined from the evaluation of the lubricin analysis were classified into three groups according to each sample's localisation: meniscus, *eminentia intercondylaris*, and tibial plateau. Because these values were normally distributed (Shapiro-Wilk test [40]) for each group, the data were averaged and the differences between the three localisations were determined by an ordinary one-way ANOVA with an additional uncorrected Fisher's LSD test.

In all cases, a significance level was set at p<0.05.

## 3 Results

### 3.1 Friction study

**Fig. 4:**
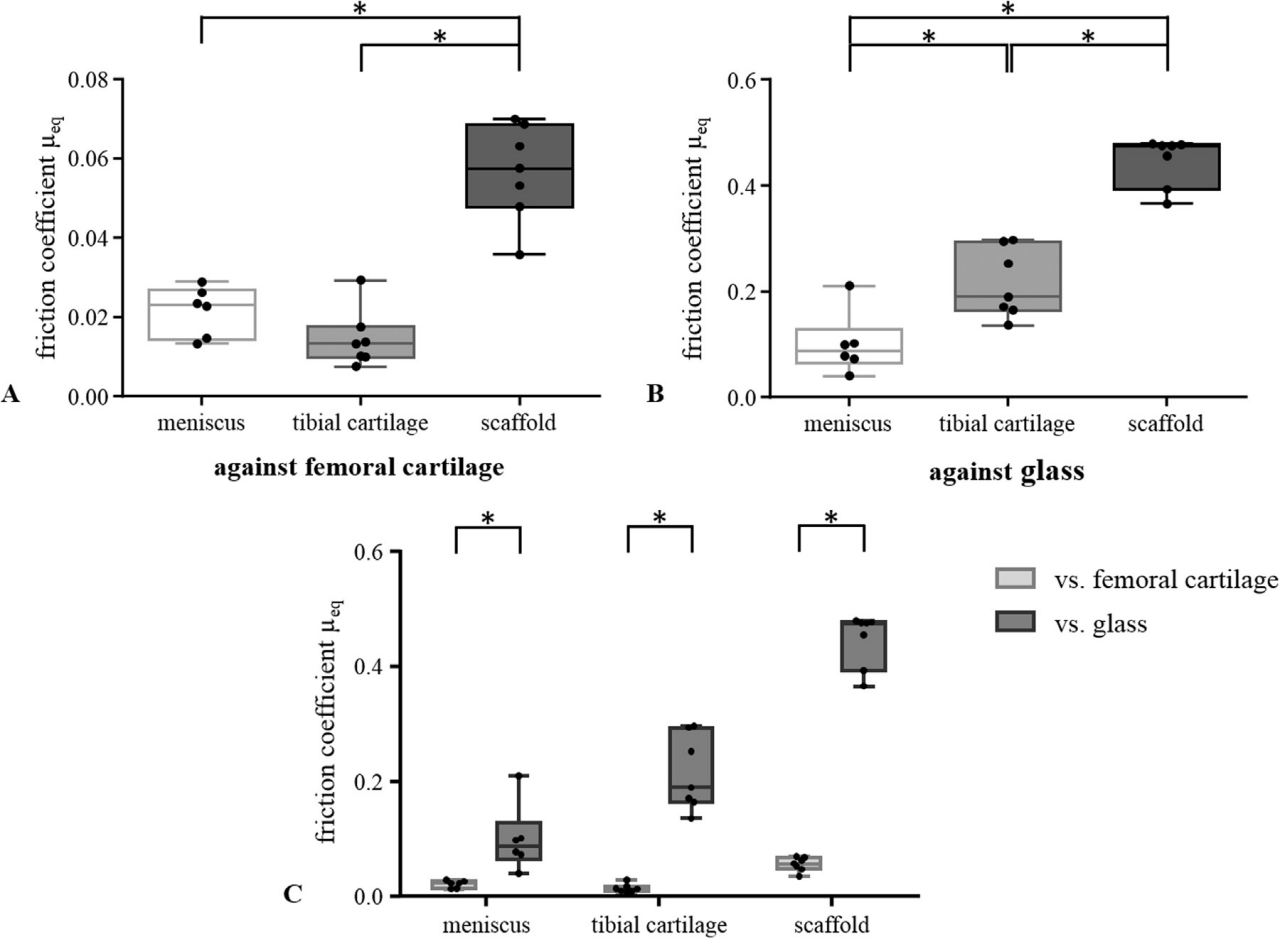
Box plot with median, minimum and maximum values of the equilibrium friction coefficient *µ*_*eq*_ of all cylindrical samples (meniscus, tibial cartilage and the meniscal scaffold) obtained during testing against the femoral cartilage sample (A) and glass (B) and their comparison (C). *p<0.05 n=7.

The silk fibroin scaffold reached an equilibrium friction coefficient *µ*_*eq*_ when sliding against femoral cartilage of 0.056±0.012, which was significantly higher in comparison to the physiologically articulating surfaces of both the meniscus against cartilage (*µ*_*eq*_=0.021±0.006, p<0.0001) and the cartilage against cartilage (*µ*_*eq*_=0.014±0.007, p<0.0001) (Fig. 4, A). Using glass as the opposing surface, the *µ*_*eq*_ of all material pairings were significantly higher compared to the friction coefficients obtained with the physiological counter surface (meniscus vs. glass: 0.100±0.058, p=0.0032; tibial cartilage vs. glass: 0.215±0.065, p<0.0001; silk fibroin scaffold vs. glass: 0.446±0.047, p<0.0001) (Fig. 4, C). Additionally, with the glass as opposing surface, there was a significant difference between the pairings, with the µ_*eq*_ of tibial cartilage being higher compared to meniscus (p=0.0022). In contrast, when using cartilage as the opposing surface, no significant difference was observed between meniscus and tibial cartilage (p=0.1815) (Fig. 4, B).

For the tibial cartilage samples, the *µ*_*eq*_ increased most by almost 15 times when using glass as the opposing surface in comparison with cartilage, while for the meniscus and the replacement scaffold this increase with glass was approximately 4.5 and 8 fold higher, respectively.

**Fig. 5:**
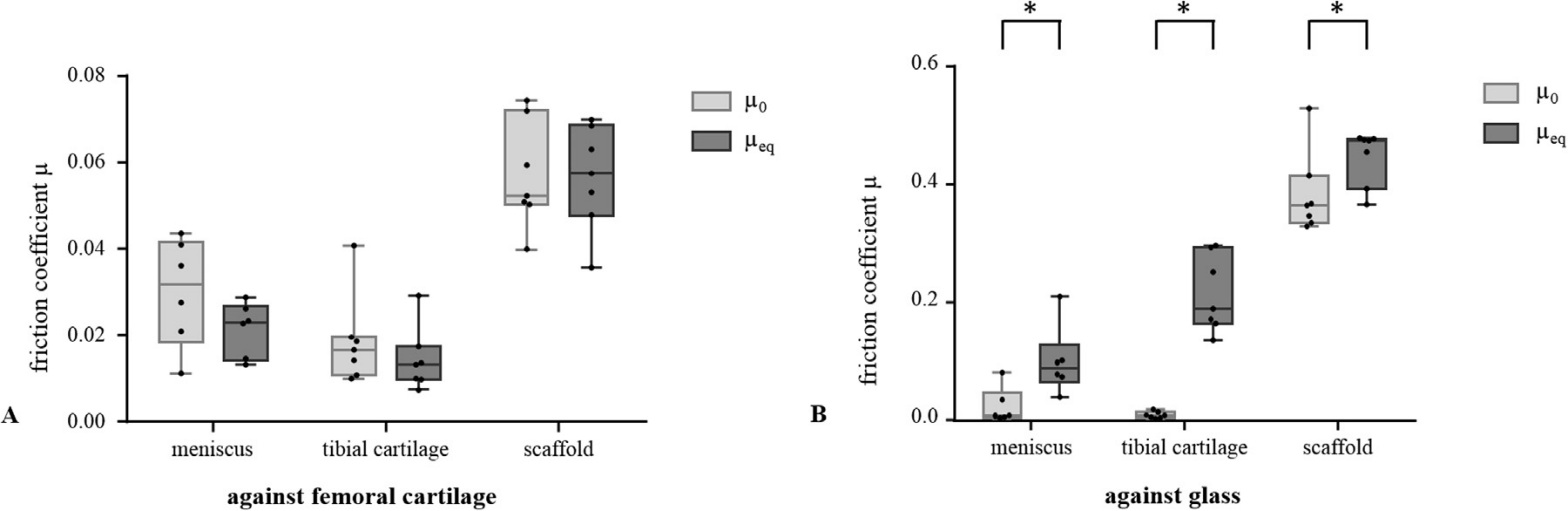
Box plot with median, minimum and maximum values of the friction coefficient determined at the onset of the test *µ*_*0*_ in comparison to that obtained after reaching the equilibrium *µ*_*eq*_ with femoral cartilage (A) and glass (B) as the counter surfaces. *p<0.05, n=7.

The three cylindrical samples revealed no significant changes with time in comparison with the friction coefficient *µ*_*0*_ determined at the experimental onset when tested against femoral cartilage (meniscus: *µ*_*0*_=0.030±0.012, *µ*_*eq*_=0.021±0.006, p=0.060; tibial cartilage: *µ*_*0*_=0.019±0.010, *µ*_*eq*_=0.014±0.007, p=0.293; silk fibroin scaffold: *µ*_*0*_=0.057±0.012, *µ*_*eq*_=0.056±0.012, p=0.906) (Fig. 5, A). In contrast, their equilibrium friction coefficient *µ*_*eq*_ of was significantly increased in comparison to the initial friction coefficient *µ*_*0*_ when tested against glass (meniscus: *µ*_*0*_=0.023±0.030, p=0.0056; tibial cartilage: *µ*_*0*_=0.009±0.006, p<0.0001, silk fibroin scaffold µ_0_=0.384±0.070, p=0.0131) (Fig. 5, B).

**Fig. 6:**
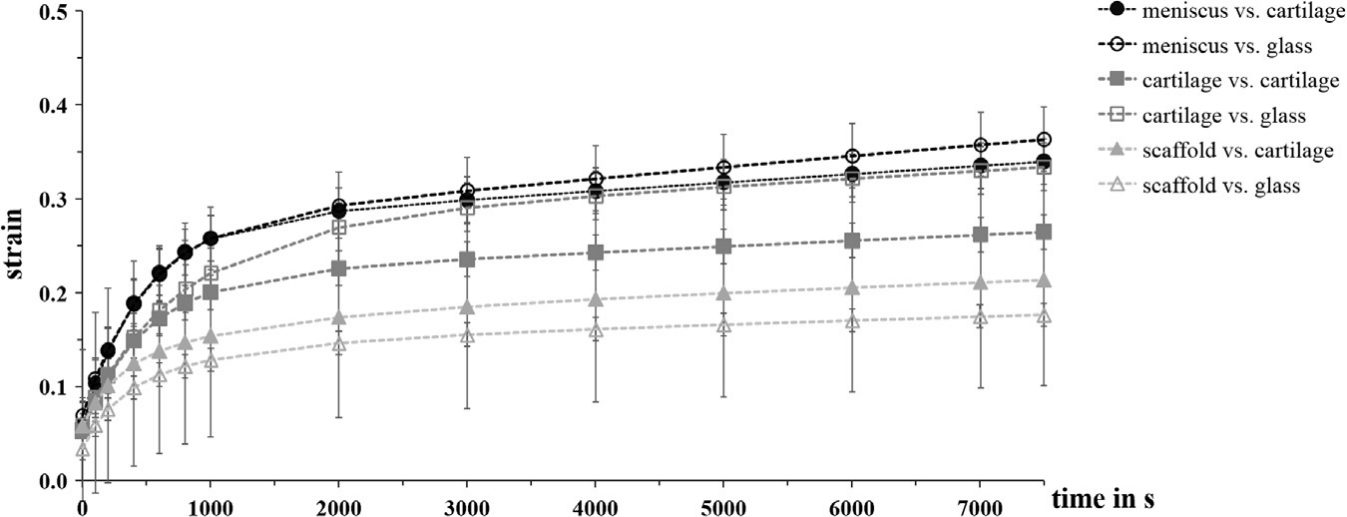
Strains of all cylindrical samples (mean±standard deviation) tested against the flat femoral cartilage samples (filled data points) and glass (empty data points). All strain values displayed an increase with time until an equilibrium was reached, which was typical for an unconfined creep configuration.

The strains of all the cylindrical samples displayed an increase over time until an equilibrium was reached, which is a typical behaviour for biphasic materials in an unconfined creep configuration (Fig. 6). With regard to the three types of cylindrical sample, the equilibrium strain value *ε*_*eq*_ was lowest of the scaffold at 0.269±0.178 and 0.219±0.133 when tested against femoral cartilage and glass, respectively. The highest strain level at equilibrium (*ε*_*eq*_) was attained by the meniscus samples at 0.339±0.042 and 0.363±0.034 when tested against femoral cartilage and glass, respectively. Furthermore, the strain values obtained when testing against femoral cartilage on day 1 and against glass on day 2 did not differ significantly (meniscus: p=0.247, tibial cartilage: p=0.200, scaffold: p=0.121).

### 3.2 Lubricin analysis

**Fig. 7:**
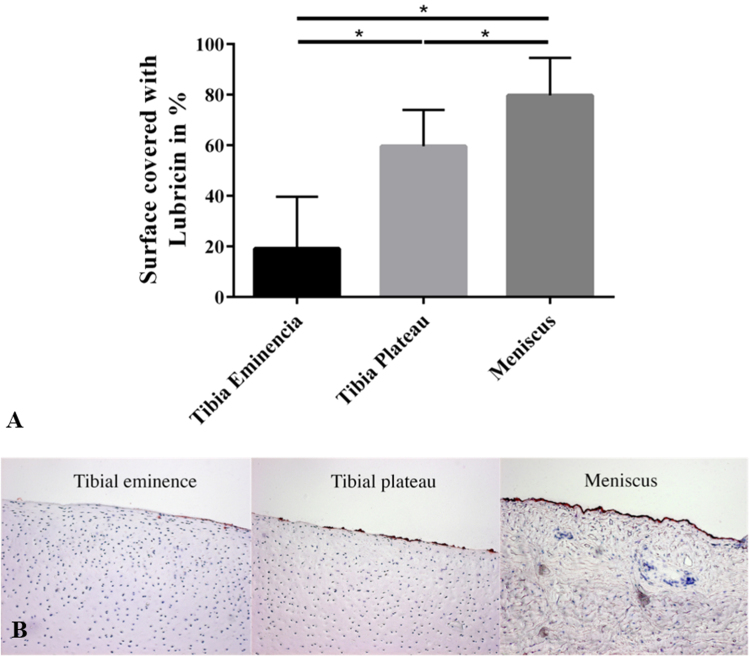
Mean percentage and standard deviation of the lubricin coverage of the tissue surface of the meniscus (n=10), tibial plateau (n=6) and tibial eminence (n=5) covered with lubricin; *p<0.05 (A) and a representative histological slide of each surface (B). Lubricin is stained in red. (For interpretation of the references to color in this figure legend, the reader is referred to the web version of this article).

Approximately 80% of the meniscus surface was covered with lubricin (Fig. 7, A). Therefore, the superficial lubricin deposition on meniscus was significantly higher than on the cartilage samples from the medial tibial plateau (p=0.0266) and the *eminentia intercondylaris* (p<0.0001). The least lubricin was found at the tibial eminence, where <20% of the surface was positively stained (Fig. 7, B).

## 4 Discussion

The silk fibroin scaffold tested in the current study exhibited equilibrium friction coefficients that were significantly different to that of physiologically articulating surfaces when tested against articular cartilage. This refutes the first hypothesis. However, the resultant friction coefficient after >2 h testing (*µ*_*eq*_=0.056) was still within the range of the basic requirements of tribological properties for meniscal substitutes, further elaborated by Rongen et al. [13]. Within these requirements, the friction coefficient is proposed to be as similar as possible to those of the meniscus and to be ≤0.05. However, testing against glass, which was typically performed in most previous friction studies [19], [22], [25], [28], [32], led to a significant increase in the friction coefficient of meniscus (µ_eq_=0.100), articular cartilage (*µ*_*eq*_=0.215) and silk fibroin scaffold (*µ*_*eq*_=0.446). Similar high friction coefficients were also demonstrated by Galley et al. for a polyurethane scaffold as a meniscal replacement when tested against glass (*µ*_*eq*_≅0.5) [32].

The large differences between the friction coefficients when testing against femoral cartilage and glass demonstrated in the current study are most likely attributable to the lack of pressurisation of the interstitial fluid in both non-biological tissues (i.e. the silk fibroin scaffold and the opposing glass surface) [22]. Previous studies have shown that the friction coefficient of cartilage increases with time when tested against a non-biologic material like glass from a mean of *µ*_*0*_=0.01 to *µ*_*eq*_=0.25 [19], [22], [23], [24], [25], [32]. This phenomenon results from the fluid load support, which decreases with time from almost 100% at the test onset to almost 0% once equilibrium is reached [19], [20], [22], [34]. Assuming the tissue that bears the load is responsible for the friction, it becomes clear that the fully pressurised fluid creates the observed low friction coefficient at the time of load application. During the creep process, a continuous transfer of the load from the fluid to the solid matrix occurs with full support provided by the solid matrix after reaching equilibrium. Hence, in this state, the solid matrix is mainly responsible for the increased friction coefficient. Therefore, provided at least one of the opposing surfaces is of cartilaginous tissue, a low initial friction coefficient is achieved, which is not the case for scaffold against glass.

Nevertheless, the friction coefficient of tibial cartilage determined in the current study against glass (*µ*_*eq*_=0.215±0.065) fits well to previously published value ranges [19], [22]. This in turn emphasises the validity of the friction-testing apparatus and therefore of the results in the present study.

Interestingly, the friction coefficient resulting when cartilage was the opposing surface was always significantly less in comparison with glass as the opposing surface, which confirmed the second hypothesis. In addition, it emphasises the importance of choosing appropriate articulating surfaces to obtain relevant friction coefficients for joints. This in particular applies to the characterisation of the frictional behaviour of materials for meniscal replacement to assess whether future implants also ensure a long-term chondroprotective function.

In contrast to the friction coefficient determined when glass was the opposing surface, no increase in friction with time could be detected when the tests were performed against cartilage. This was true for both the biological tissues, cartilage and meniscus, and also for the silk fibroin scaffold. The main reason for the constant low friction was the choice of the opposing surface. The testing configuration of a flat opposing cartilage surface sliding against a constantly loaded pin allows a cyclic loading of the flat cartilage sample. While the tissue in the uSH was permanently loaded, the contact area on the opposing flat cartilage sample moved along the sample and was, therefore, loaded only for the time the upper cylindrical sample needed to pass one sample diameter. This enabled the different regions of the flat cartilage samples to recover before the pin loaded that region again and consequently no creep occurred. Due to the biphasic ultrastructure of the biological samples, the fluid phase supported the applied load during the entire experiment. Shi et al. and Caligaris et al. both demonstrated a similar constant, low friction coefficient with time for a cartilage-on-cartilage testing configuration [19], [21]. Shi et al. tested an aluminium pin against a cartilage disc (AC configuration) and no increase in friction with time was detectable, as well. This AC configuration is comparable to the scaffold against cartilage configuration we tested in the present study, whereby no increase in the friction was similarly observed. In addition, Shi et al. estimated the corresponding fluid load support, which remained >80% during the experiment. This result supports the evidence of the fluid pressurisation theory and specifically it's important role in friction studies.

The meniscus samples also displayed an increase in friction with time when tested against glass, but reached a significantly reduced equilibrium friction value compared to articular cartilage. The meniscus and articular cartilage vary considerably in their extracellular matrix composition, in particular, the type of collagen and the amount of proteoglycans. While the articular cartilage is composed of collagen type II, the meniscus mainly consists of collagen type I and has only 10% of the proteoglycan content of articular cartilage [1], [2], [3], [4]. However, when considering the friction properties, it is logical to pay closer attention to the tissue's surface properties. Therefore, we additionally assessed the lubricin content on the surface of the bovine meniscus, the articular cartilage from the *eminentia intercondylaris* and from the tibia plateau. We found that 80% of the meniscus surface was covered with lubricin. In contrast, only 60% of the tibia plateau and 20% of the *eminentia intercondylaris* were covered with lubricin. These results correlate with the lower friction coefficient of the meniscus compared to articular cartilage observed in the current study (Fig. 5, B).

### 4.1 Limitations

The test setup was designed according to a *pin-on-plate* configuration. Therefore, flat cartilage samples had to be harvested from the three-dimensionally convex shaped femoral condyles. Consequently, any small unevenness in the surface of the flat cartilage may have affected not only the friction force but also the strain measurements. However, the displacement signal of each cylindrical sample determined by the contactless laser distance sensor LS and the resultant strain values displayed no statistical difference when tested against cartilage or the smooth glass surface (p≥0.15).

### 4.2 Conclusion

The current study presents for the first time a characterisation of the frictional properties of a meniscal replacement material. The friction coefficient of a scaffold based on silk fibroin was higher compared to the physiologically articulating surfaces, but still within the range of the basic requirements for meniscal substitutes [13]. Whether this enables silk fibroin scaffold to provide a long-term chondroprotective function has to be confirmed *in vivo*. Interestingly, it could also be shown that glass as the opposing surface is not appropriate for friction testing of implants, as it produces significantly higher friction coefficients than occurring in the physiological environment. Glass may be useful to perform comparative studies on different biomaterials. However, investigating physiologically relevant friction coefficients occurring in a synovial joint requires the use of cartilage as the opposing surface.
